# A systematic review and meta-analysis of operative versus non-operative management for first time traumatic anterior shoulder dislocation in young adults

**DOI:** 10.1177/17585732241254693

**Published:** 2024-05-20

**Authors:** Joseph Cutteridge, Joe Dixon, Pierre Garrido, Nicholas Peckham, Carolyn Smith, Alex Woods, Stephen Gwilym

**Affiliations:** 1 Nuffield Department of Orthopaedics, Rheumatology and Musculoskeletal Sciences, Nuffield Orthopaedics Centre, Headington, Oxford, UK; 2 York and Scarborough Teaching Hospitals NHS Foundation Trust, York Hospital, Clifton, York, UK; 3 6831 Surrey and Sussex Healthcare NHS Trust, East Surrey Hospital, Redhill, UK; 4 97467 Bodleian Health Care Libraries, Education Centre, Horton Hospital, Banbury, UK; 5 6397 Oxford University Hospitals NHS Foundation Trust, John Radcliffe Hospital, Headington, Oxford, UK

**Keywords:** traumatic anterior shoulder dislocation, re-dislocation rate, systematic review and meta-analysis‌

## Abstract

**Background:**

The most appropriate management following primary traumatic anterior shoulder dislocation in young adults is unclear. This systematic review and meta-analysis evaluated operative versus non-operative management. The primary outcome measure was re-dislocation rate, in contrast to the often reported ‘recurrent instability’, which includes subjective instability.

**Methods:**

Our review was prospectively registered with PROSPERO (CRD42022322600) and reported as per PRISMA guidelines. Selection criteria included mean age of participants between 15 and 25 and minimum follow-up of 1 year.

**Results:**

21 studies meet the inclusion criteria with 5142 patients included. The mean age of patients was 23, with 87% male. There was a median of 54 patients per study and a mean follow up of 46 months per study. The mean re-dislocation rate was 16.08% in the operative group and 24.84% in the non-operative group. In the subgroup meta-analysis, including only RCTs, comparing arthroscopic stabilisation vs non-operative there was an odds ratio of 0.09, strongly favouring intervention.

**Discussion:**

This systematic review found the literature available supports surgical intervention in patients under the age of 25, in order to reduce re-dislocation. However, there is a lack of cost-effectiveness data to support these findings, and this should be an area of future research.

## Introduction

The shoulder is the most frequently dislocated major joint in the body.^1^ Over 95% dislocate anteriorly and occur following traumatic events such as falls or sporting injuries. Around 50% of dislocations occur in patients aged 15–29 years.^1,2^

Following primary traumatic anterior shoulder dislocation young adults have a much higher chance of re-dislocation than the rest of the population.^3,4^ The British Elbow and Shoulder Society (BESS) state that the risk of recurrent instability is inversely proportional to the age at dislocation, with males under the age of 20 years having approximately 72% chance of recurrent instability.^3,5^ The distinction between “re-dislocation” and “recurrent instability” is a subtle but important one for patient counselling and the terms have often, but incorrectly, used interchangeably.

Every dislocation carries the risk of vascular or nerve injury, most often the axillary nerve, and rotator cuff tears.^6^ Patients are also at risk of structural lesions, including Hill-Sachs and Bankart lesions, which themselves predispose to further dislocations.^7–9^ In the long-term, recurrent dislocation increases the risk of arthritic changes in the shoulder joint, with the number of instability events exhibiting linear correlation with the likelihood of post-traumatic arthritis.^10–12^ Thus, intervention to limit the development of structural lesions and prevent recurrent dislocations is important for long-term shoulder health, as well as patient quality of life.

The most cost-effective treatment of primary traumatic anterior shoulder dislocation in young adults, after initial reduction, is currently debated within the medical literature. Current approaches vary from early mobilisation to surgical interventions, both open and arthroscopic.^13^ Surgery is utilised because the perceived reduction in recurrent dislocation is seen to outweigh the increased costs and potential complications, as compared to conservative management. BESS currently states that primary arthroscopic repair should be considered in first-time dislocators aged <25 years, due to the high risk of recurrent dislocation.^5^

The objective of this systematic literature review (SLR) is to elucidate how primary traumatic anterior shoulder dislocation among those aged 15–25 years should be managed definitively to give the best patient outcomes, using re-dislocation rate as our primary outcome, with secondary outcomes including return to sport rate and Patient Reported Outcomes Measures (PROMS).

Importantly, this review separates literature pertaining to “re-dislocation” from symptoms of instability.

## Materials and methods

### Search strategy

**Figure 1.**
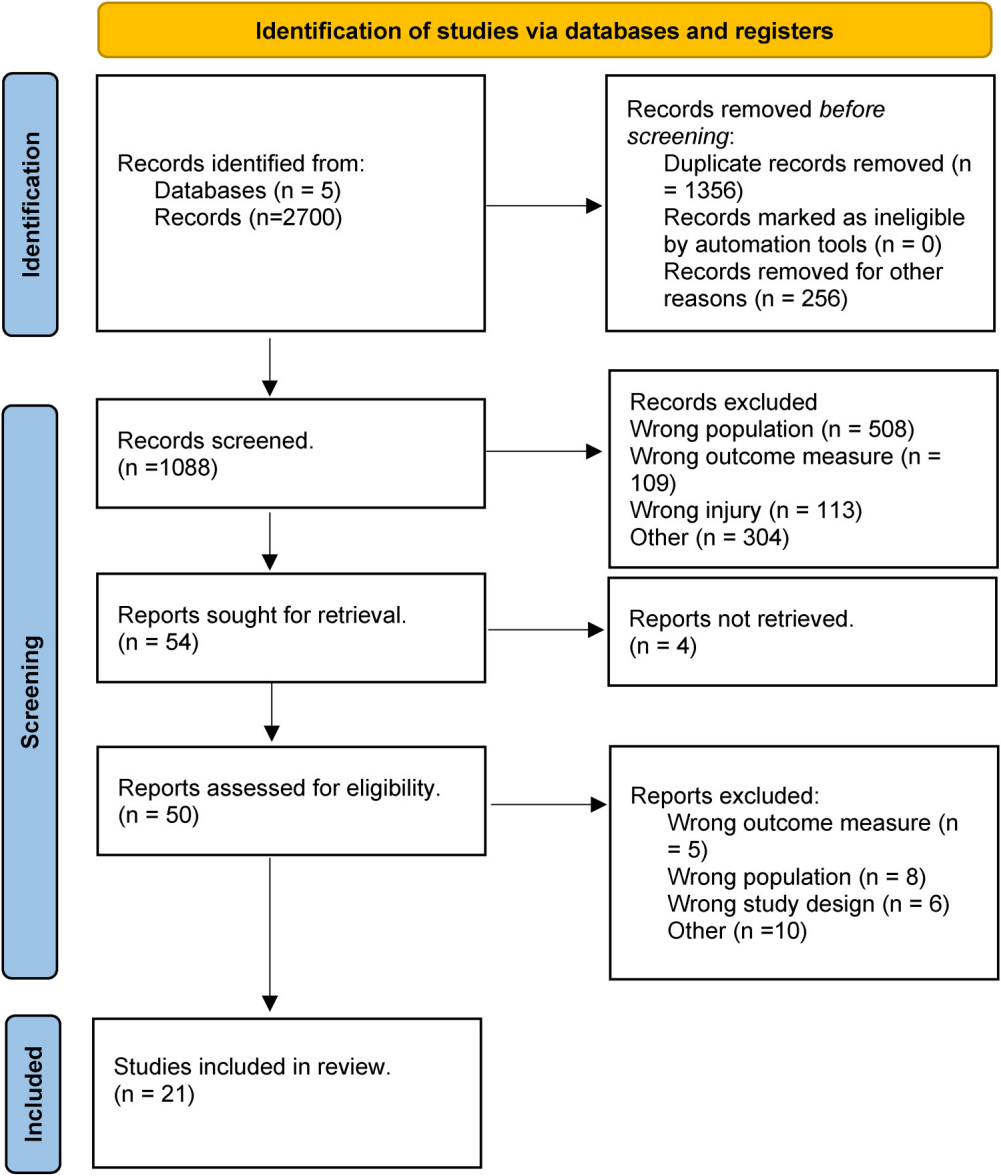
PRISMA flow diagram including record identification, screening and selection.

This systematic review was conducted using guidance from the Joanna Briggs Institute.^14^ Findings are reported according to the extension for Preferred Reporting Items for Systematic Reviews and MetaAnalyses (PRISMA) guidelines,^15^ with our summary PRISMA flow diagram shown below (Figure 1). Prior to commencing this review, a study protocol was developed and registered with PROSPERO (CRD42022322600).

Our search strategy was designed in collaboration with an information expert and consisted of a comprehensive search of PubMed, MEDLINE, EMBASE, Scopus, web of science, Cochrane Library, Google Scholar databases and grey literature. All the databases were searched separately, with similar but adapted search strategies and we have included each database search strategy on the Search Strategy spreadsheet (Supplemental Table 1). All search strategies were devised by the information expert for this systematic review only and have not been used previously elsewhere. Duplicates were removed using EndNote 20 deduplication function, with each set of duplicate results carefully examined to ensure that false hits were not removed.

### Selection criteria

Studies were included if they met the following criteria: (1) assessment of primary anterior traumatic shoulder dislocation; (2) reporting of re-dislocation rate; (3) minimum 1 year follow up; (4) mean age between 15 and 25 years. Exclusion criteria were as follows: (1) assessment of recurrent shoulder dislocation; (2) assessment of non-traumatic shoulder instability; (3) level 3/4/5 evidence (cross-sectional studies, case study/series, expert opinion pieces, cadaveric and biomechanical studies, and bench research) and SLRs analysing such evidence; (4) non-English language; (5) conference abstracts.

### Data extraction

All papers identified in our search underwent screening by a minimum of two authors. This initially consisted of title and abstract screening using the Rayyan.AI software, followed by a full text review of potential studies to ensure they met our selection criteria. Included studies were extracted for the following data: last name of first author, year of publication, journal, study design, total number of patients, demographic data (age and sex), number of patients lost to follow up, length of follow up, inclusion/exclusion criteria, primary outcome, return to sport rate and PROM scores (if available), number of treatment groups, interventions used. Data were inputted into a shared Google Sheet and any cases of disagreement were checked for with a third author.

### Risk of bias

Randomised control trials were evaluated using the Cochrane risk-of-bias tool for randomised control trials (RoB2).^16^ Non-randomised studies were evaluated using the methodological index for non-randomised studies (MINORS) criteria.^17^

### Statistical analysis

Data for PROMS and return to sport rate were evaluated by calculating a mean and standard deviation (SD) within each intervention arm of every study in which they were reported. The most widely PROM used scoring systems were Western Ontario Shoulder Instability Index (WOSI) and the Rowe score. Pooled effect sizes of recurrent dislocation were estimated by calculating the mean re-dislocation rate for each intervention arm of every included study, which was then weighted by sample size.

For the purposes of meta-analysis of our primary outcome, recurrent shoulder dislocation, we utilised the Review Manager (RevMan) V5.4 software program. Meta-analysis of studies directly comparing operative versus non-operative interventions was devised. A subgroup analysis of RCTs evaluating arthroscopic stabilisation versus non-operative management was also conducted. This was undertaken because arthroscopic stabilisation is the recommended surgical intervention in this population in the current BESS guidelines.^5^ RCTs were selected in order to isolate the highest form of evidence and provide the most meaningful results. Due to the expected heterogeneity between studies, stemming from differences in local surgical protocols, individual surgeon's preferences etc., a random effects model was employed. Absolute numbers of patients and events are presented for each trial. The summary statistics is presented as odds ratios (OR) and their corresponding 95% confidence intervals are shown. For each analysis, τ2 is presented as an estimate for the variance of true treatment effects between the trials, and the I^2^ used to display the estimated proportion of variability that can be attributed to trial heterogeneity. However, the authors recognise the uncertainty in the I^2^ measure; therefore, we have avoided using simple thresholds to diagnose heterogeneity.^18^ A two-tailed significance level of 5% will be used for all statistical analyses. The overall certainty of evidence for the primary outcome will be assessed in accordance with the Grading of Recommendations, Assessment, Development and Evaluation (GRADE) approach.^19^

## Results

Following review, 21 studies met inclusion criteria (Table 1). The risk of bias assessment for the seven included RCTs revealed that all but one study was at low risk of bias (Supplemental Table 2 & Supplemental Figure 1). One study was deemed at high risk of bias due to its method of randomisation, which was based on the last digit of each patient's social security number. This methodology, whilst outdated, was not considered to be a serious flaw in experimental design. Furthermore, this study had otherwise robust methodology and so the paper was included in our analysis. The risk of bias for non-randomised studies highlighted that many did not explicitly state certain characteristics of study design, with 23% of specified MINORS criteria unreported across all papers (Supplemental Table 3 & Supplemental Figure 2). However, no other study was found to be at high risk of bias. Thus, with all factors considered, we consider that our analysis is at low risk of bias.

**Table 1.** Table of studies included in systematic review.

First author	Year	Journal	Study design	Number of patients	Follow up duration in months	Group 1 description	Group 1 re-dislocation rate (%)	Group 2 description	Group 2 re-dislocation rate (%)
Jakobsen^20^	2007	Arthroscopy	Randomised control trial	76	120	Open Bankart repair	9	Sling	62
Kirkley^21^	2005	Arthroscopy	Randomised control trial	40	79	Anterior stabilisation	18.75	Immobilisation for 3 weeks followed by rehabilitation	60
Hovelius^22^	1996	Journal of Bone and Joint surgery (AM)	Randomised control trial	24	120	Immobilisation	48	Sling	49.5
Kim^23^	2011	International Orthopaedics	Cohort	110	24	Arthroscopic repair	2	NA	-
Larrain^24^	2001	Arthroscopy	Prospective non-randomised trial	46	67	Arthroscopic repair	4	Immobilisation for 2–4weeks	95
Maeda^25^	2002	Journal of Orthopaedics Science (JP)	Case control	79	24	Immobilisation for 3 weeks	85	Immobilisation for 4–7 weeks	69
Arciero^26^	2001	American Journal of Sports medicine	Cohort	57	37	Arthroscopic repair	12	NA	-
Bottoni^27^	2002	American Journal of Sports medicine	Randomised control trial	24	24	Arthroscopic repair	11.7	4 weeks immobilisation	75
Te slaa^28^	2003	Journal of Elbow and Shoulder surgery	Cohort	31	60	Diagnostic arthroscopy and washout	55	NA	-
Finestone^29^	2009	Journal of Bone and Joint surgery (BR)	Case-control	51	35	15-degree external rotation immobilisation	37	Bracing internal rotation	41
De carli^30^	2019	International Orthopaedics	Case-control	160	82	Arthroscopic repair	14	2 weeks immobilisation, abduction/ internal rotation, 2–6 weeks	71
Gigis^31^	2014	Journal of Paediatric Orthopaedics	Case-control	72	36	Arthroscopic stabilisation	13	Sling immobilisation and physio	70
Wintzell^32^	1999	Journal of Shoulder & Elbow surgery	Prospective randomised trial	30	24	Arthroscopic lavage	20	Non operative	60
Pouges^33^	2021	The American Journal of Sports Medicine	Prospective randomised trial	40	24	Arthroscopic Bankart repair	0	3 weeks immobilisation in internal rotation	60
Uhring^34^	2014	Orthopaedics and Traumatology research	Cohort	31	24	Arthroscopic Bankart repair	0	4 weeks immobilisation in internal rotation	71
Whelan^35^	2014	Clinical Orthopaedics and Related Research	Randomised control trial	60	25	4 weeks external rotation immobilisation	22.2	4 weeks internal rotation immobilisation	32
Wheeler^36^	1989	Arthroscopy: The Journal of Arthroscopic and Related Surgery	Cohort	47	14	Arthroscopic repair	22	Non-operative	81.6
Shih^37^	2011	Formosan Journal of musculoskeletal disorders	Cohort	67	72	Arthroscopic repair	5.1	Sling immobilisation for 4	92
Robinson^4^	2006	Journal of Bone and Joint surgery (AM)	Cohort	284	12	Sling immobilisation	53	NA	-
Rees^38^	2019	Health Technology assessment	Cohort	3759	47	Surgical intervention (unspecified)	20	Non operative (unspecified)	20
Mcleod^39^	2020	Irish Journal of Medical Sciences	Cohort	54	24	Arthroscopic Bankart repair	21	NA	-

The total number of patients included was 5142, with a median of 54 patients per study, and a mean follow up of 46 months. The mean age of patients in the study was 23 years, and 87% of patients were male.

The mean re-dislocation rate was 16.08% in the operative group and 24.84% in the non-operative group. The return to sport rate was 85.24% in the operative group and 78.34% in the non-operative group. The relative risk reduction in the operative group versus the non-operative group was 34.26% (22.00–44.60%), the absolute risk reduction was 8.4% (95% CI [5.4, 11.31]), and the number needed to treat was 11.93 (95% CI [8.84, 18.34]).

Open surgery had the lowest overall re-dislocation rate (9%), however this was only reported in one paper (Jacobsen et al. 2007), which had a low sample size of 37 patients. Arthroscopic stabilisation had a mean re-dislocation rate of 10.76% and a return to sport rate of 87.53%. The largest included study did not detail the specific operations conducted within its operative arm,^38^ reporting an operative re-dislocation rate of 20%, resulting in our overall re-dislocation rate for operative interventions coming to 16.08%.

The mean Rowe score was 89.33 in the arthroscopic stabilisation group and 54.03 in the non-operative group, with a significant mean difference of 35.30 (*p*-value: 0.019). There was no statistically significant difference between WOSI scores, 189 in the operative group and 184.85 in the non-operative group, with a mean difference of 4.15 (*p*-value: 0.792).

**Figure 2.**
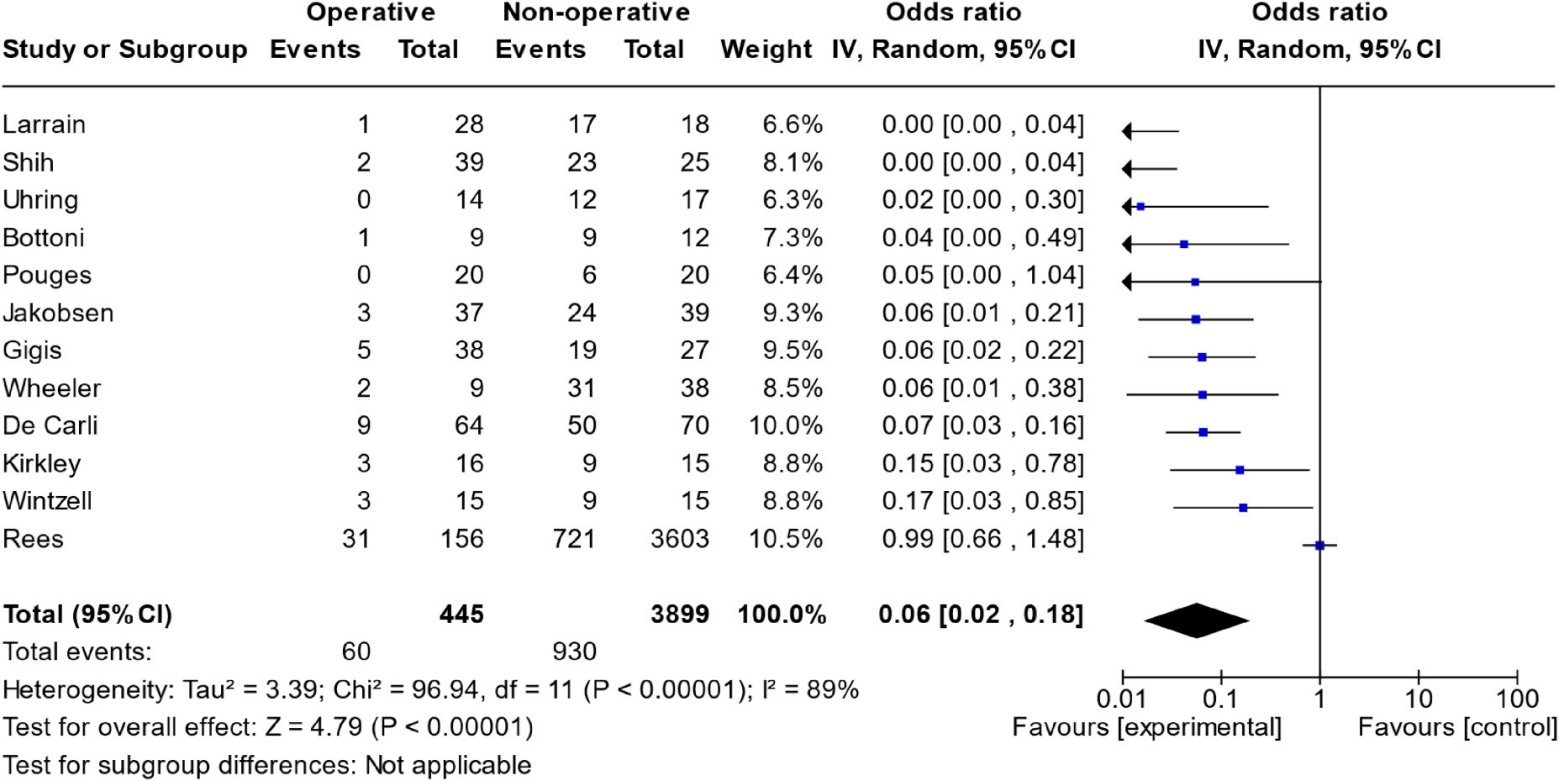
Meta-analysis of operative versus non-operative management.

**Figure 3.**
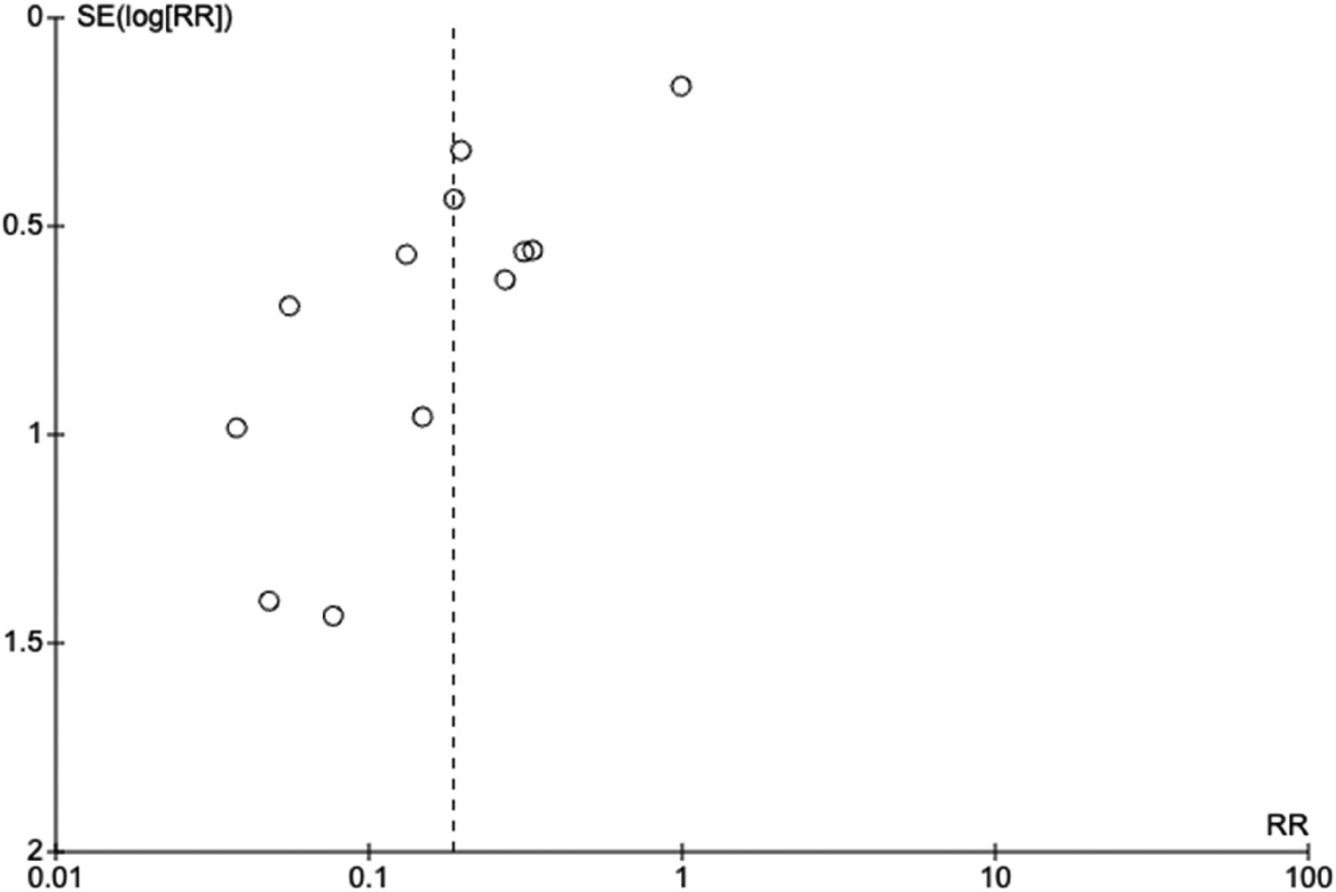
Funnel plot of studies evaluating operative versus non-operative management.

The meta-analysis of all studies that directly compared operative versus non-operative can be seen in Figure 2. The total effect measure was an OR of 0.06 (0.02–0.18, *p*-value <0.00001). However, this analysis suffered from significant heterogeneity between studies, with an I^2^ of 89%. A funnel plot of this analysis is shown in Figure 3.

CI: confidence interval.

**Figure 4.**
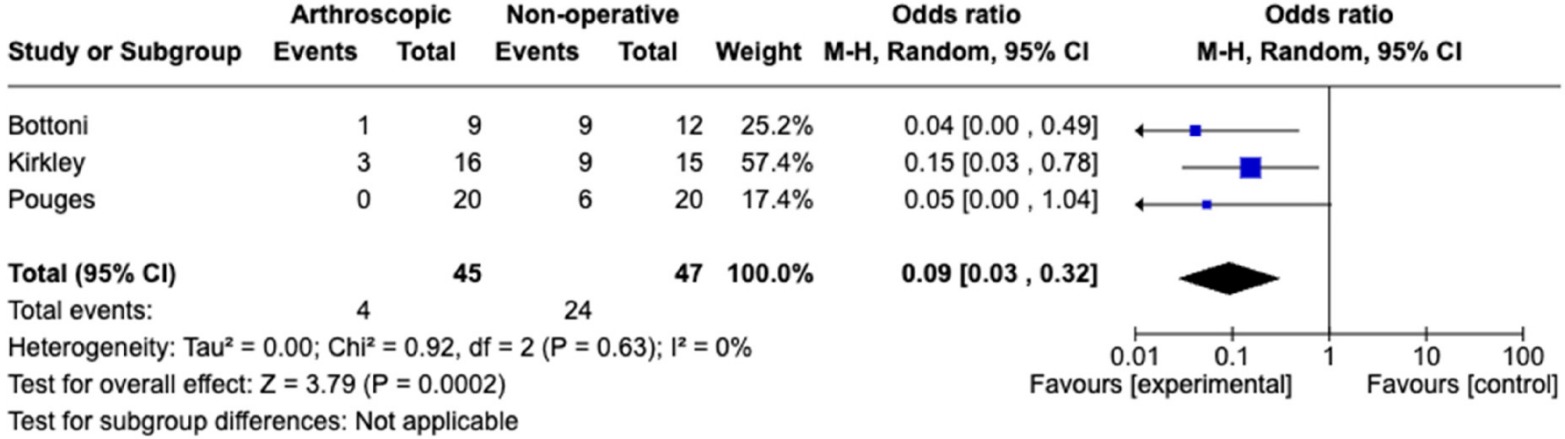
Meta-analysis of RCTs for arthroscopic repair versus non-operative management.

The subgroup analysis of RCTs comparing arthroscopic stabilisation versus non-operative management yielded three studies, as shown in Figure 4. This included 45 patients randomised to arthroscopic stabilisation and 47 randomised to non-operative management. The re-dislocation rate in the arthroscopic stabilisation group was 8.89%, whilst in the nonoperative group it was 51.06%. This yielded an OR of 0.09 (CI 0.03–0.32, *p*-value 0.002). This analysis had minimal heterogeneity, with an I^2^ of 0%.

CI: confidence interval.

**Figure 5.**
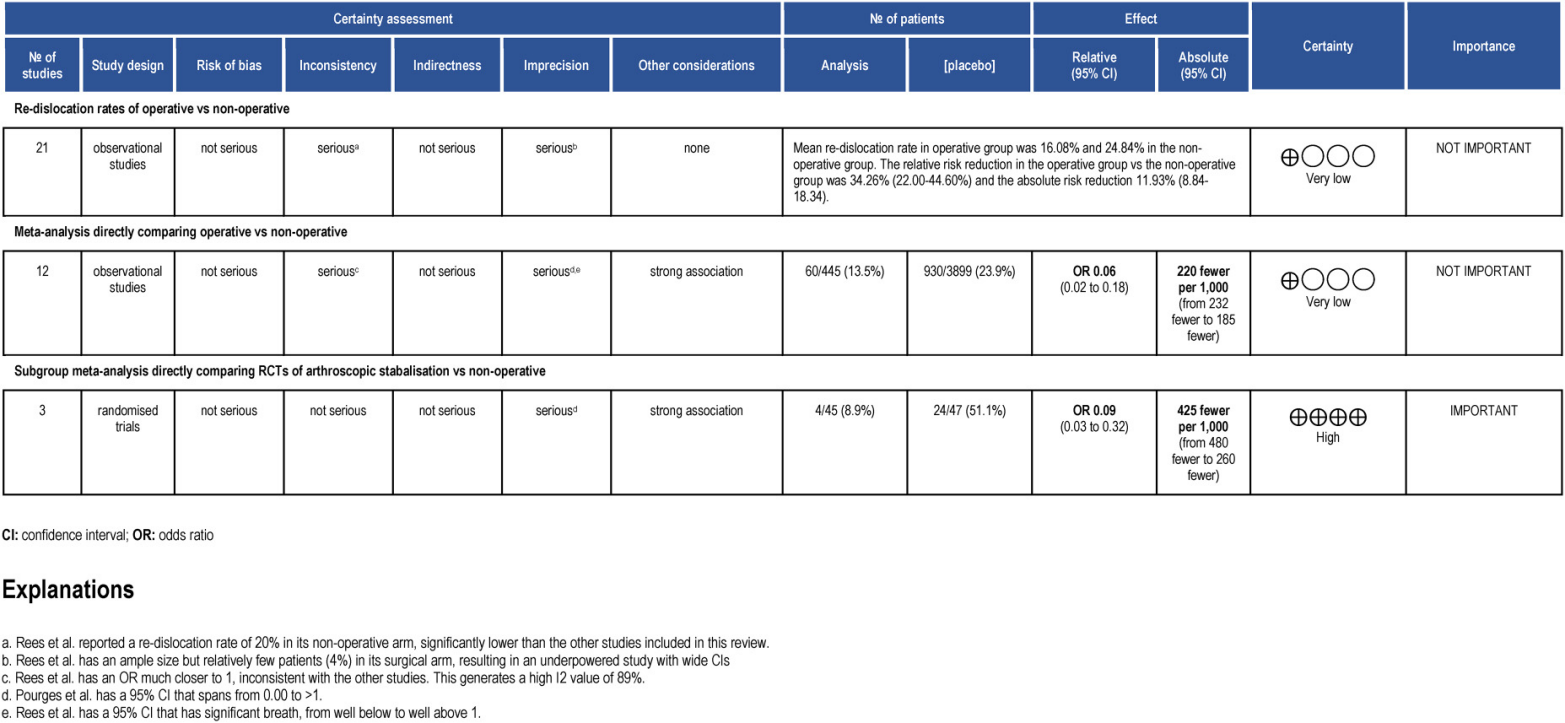
Summary of findings table, in accordance with GRADE approach. The study design reflects which type of study formed the majority within that analysis model.

The overall Summary of Findings (SoF) table can be seen in Figure 5.

## Discussion

This study describes systematic review and meta-analysis of operative versus non-operative management for first time traumatic anterior shoulder dislocations in patients aged 15–25 years of age. In all results, operative intervention reduces the risk of recurrent dislocation, when compared to non-operative management, however there is high heterogeneity in the data. Operative management also demonstrated superior return to sport rates and patient reported outcomes.

The subgroup analysis of RCTs directly comparing arthroscopic stabilisation versus non-operative (Figure 4) isolates the highest level of evidence of the most common treatment options in practice currently. The results indicate that arthroscopic stabilisation is greatly protective against subsequent shoulder dislocation, with an OR of 0.09 (CI 0.03–0.032, *p*-value 0.0002), with interstudy variability (I^2 ^= 0%). This provides further evidence to support the BESS guidelines that arthroscopic stabilisation should be considered for first-time dislocator's under 25 years.^5^ However, it should be acknowledged there were only three RCTs with low patient numbers included in this analysis which limits the certainty we have in these findings.

The re-dislocation rates presented in this review differ significantly from previously described rates of recurrent dislocation, previously cited to be around 70%, rising to 86% in young males.^4,5^ Our reported re-dislocation rates of 16.08% in operative patients and 25.03% in non-operative patients are such because we have separated true ‘re-dislocations’ and ‘reports of subjective instability without dislocation’.

There are important limitations of this study to consider, firstly we have not collected any data on operative complications which may influence the decision-making process. We have additionally not collected any data on the cost effectiveness of either intervention, or data on the biopsychosocial impact that recurrent dislocation may have on young adults during their formative years.

When reviewing the combined data of all included studies, an important consideration is the data for most patients (72%) came from one study,^38^ which was a national retrospective cohort study, conducted using data obtained from two computerised NHS databases (CPRD and HES). This study reported a re-dislocation rate of 20% in both its operative and non-operative arms, the latter of which is significantly lower than the other studies reported in this review. As a consequence of the large numbers in this study, this had a significant influence on our overall pooled cumulative findings, as the analysis was weighted according to study size. Removal of this study would have increased the mean dislocation rate in the non-operative group to 59%. This effect was minimised in the meta-analysis directly comparing operative versus non-operative treatment as the study was weighted at 10.5% because of the random effects model and the high level of intra-study variance.

The meta-analysis directly comparing operative versus non-operative treatment (Figure 2) suffered from high heterogeneity (I^2 ^= 89%), severely limiting the conclusions we can draw. This is highlighted in our SoF table, where this model is marked as yielding evidence of low certainty.

There was marked study heterogeneity in the reporting of PROMS, with a great variety of scoring systems used, and some studies not including any patient reported measures at all. A significant difference was reported between the Arthroscopic stabilisation and Non-Operative in ROWE scores but not in WOSI scores. This should be an area of interest for further research to enable a greater understanding of the patients experience of dislocation and surgical outcomes.

The authors recognise that RCTs are expensive to run and not always ethical, feasible or practical. However, in this context, where there is uncertainty in the literature, an adequately powered multi-centre RCT would provide important information regarding management for this patient group.

In conclusion, this review is the first to specifically consider re-dislocation rates, as opposed to recurrent instability, in younger adults. It has demonstrated that the available literature generally supports operative intervention following primary traumatic anterior shoulder dislocation in young adults. However, this should be interpreted with a degree of caution, due to the high degree of heterogeneity among published studies. Our subgroup analysis of RCTs evaluating arthroscopic stabilisation revealed a significant reduction in re-dislocation rates as compared to non-operative management, reaffirming current BESS guidelines.^5^ Yet only three studies were suitable for inclusion in this analysis. Due to the lack of high-quality evidence to support these findings, the authors would recommend this area requires further research in the form of an RCT.

## Supplemental Material

sj-docx-1-sel-10.1177_17585732241254693 - Supplemental material for A systematic review and meta-analysis of operative versus non-operative management for first time traumatic anterior shoulder dislocation in young adults Supplemental material, sj-docx-1-sel-10.1177_17585732241254693 for A systematic review and meta-analysis of operative versus non-operative management for first time traumatic anterior shoulder dislocation in young adults by Joseph Cutteridge, Joe Dixon, Pierre Garrido, Nicholas Peckham, Carolyn Smith, Alex Woods and Stephen Gwilym in Shoulder & Elbow
