# Supplementary material for: A systematic review and meta-analysis of operative versus non-operative management for first time traumatic anterior shoulder dislocation in young adults
Source: Shoulder Elbow. 2024 May 20;17(2):130–9. doi: 10.1177/17585732241254693 (PMC11562324; doi:10.1177/17585732241254693)

# Supplemental material

## Search Strategy

| **Database** | **Interface** | **Coverage** | **Date** | **Hits** |
| --- | --- | --- | --- | --- |
| Cochrane Central Register of Controlled Trials | https://www.cochranelibrary.com/advanced-search/search-manager | Issue 10 of 12, October 2021 | 05/10/2021 | 64 |
| Cochrane Library | https://www.cochranelibrary.com/advanced-search/search-manager | Issue 10 of 12, October 2021 | 05/10/2021 | 12 |
| Embase | OvidSP | 1974-present | 07/11/2021 | 782 |
| Medline | OvidSP | 1946-present | 07/11/2021 | 683 |
| PubMed (Related articles search based on 3 papers referenced in PICO) | PubMed |  | 15/11/2021 |  |
| Scopus |  |  | 12/11/2021 | 1,047 |
| Web of Science | Web of Science Core Collection |  | 12/11/2021 | 112 |
| **Total:** |  |  |  | **2700** |
|  |  |  |  |  |
| Excluded studies: |  |  |  | 256 |
| Duplicates |  |  |  | 1356 |
|  |  |  |  |  |
| **Total for researcher screening** |  |  |  | **1088** |
|  |  |  |  |  |
| **Excluded studies (librarian screened):** |  |  |  |  |
| Case reports | 102 |  |  |  |
| Comment | 7 |  |  |  |
| Conference abstracts | 59 |  |  |  |
| Level IV/V evidence | 54 |  |  |  |
| Non human | 1 |  |  |  |
| Non english | 33 |  |  |  |

*Supplemental table 1: Search Strategy spreadsheet*

## Risk of bias

| First Author (Year) | Adequate sequence generation | Allocation concealment | Blinding of outcome assessor | Completeness of data | Selective outcome reporting | Other potential threats to viability | Overall assessment |
| --- | --- | --- | --- | --- | --- | --- | --- |
| Jakobsen (2007) | Unclear | Low risk | Low risk | Low risk | Low risk | Low Risk | Low risk |
| Kirkley (2005) | Low risk | Low risk | Unclear | Low risk | Low risk | Low Risk | Low risk |
| Wintzell (1999) | Low risk | Low risk | Low risk | Low risk | Low risk | Low Risk | Low risk |
| Pouges (2021) | Low risk | Low risk | Unclear | Low risk | Low risk | Low Risk | Low risk |
| Whelan (2014) | Low risk | Low risk | Low risk | Low risk | Low risk | Low Risk | Low risk |
| Bottoni (2002) | **High risk** | Unclear | Unclear | Low risk | Low risk | Low Risk | **High risk** |
| Hovelius (1996) | Unclear | Unclear | Low risk | Low risk | Low risk | Low Risk | Low risk |

*Supplemental table 2: Risk of bias for RCTs using the Cochrane risk-of-bias tool for randomised control trials (RoB2).*

*Supplemental figure 1: ROB2 risk of bias criteria presented by category for all randomised studies.*

| First author (Year) | Adequate control group | Contemporary groups | Baseline equivalence of groups | Adequate statistical analysis | Clearly stated aim | Inclusion of consecutive patients | Prospective data collection | Endpoint appropriate  to study aim | Unbiased assessment  of study endpoint | Follow-up period appropriate to study  aim | <5%  loss to follow up | Prospective calculation  of study size |
| --- | --- | --- | --- | --- | --- | --- | --- | --- | --- | --- | --- | --- |
| Maeda  (2022) | 1 | 2 | 2 | 2 | 2 | 2 | 2 | 2 | 2 | 2 | 2 | 2 |
| Wheeler (1989) | 2 | 1 | 0 | 1 | 2 | 2 | 0 | 0 | 0 | 2 | 2 | 0 |
| Uhring (2014) | 2 | 2 | 2 | 2 | 2 | 2 | 2 | 0 | 0 | 2 | 1 | 0 |
| Robinson  (2006) | NA | NA | NA | NA | 2 | 2 | 2 | 2 | 0 | 2 | 1 | 0 |
| Rees (2019) | 2 | 2 | 2 | 2 | 2 | 2 | 2 | 2 | 2 | 2 | 2 | 2 |
| De Carli  (2019) | 2 | 2 | 2 | 2 | 2 | 2 | 2 | 2 | 2 | 2 | 1 | 0 |
| Shih (2011) | 2 | 2 | 2 | 2 | 2 | 2 | 2 | 2 | 0 | 2 | 2 | 0 |
| Finestone (2009) | 2 | 2 | 0 | 1 | 2 | 0 | 0 | 0 | 0 | 2 | 2 | 2 |
| Te Slaa  (2003) | NA | NA | NA | NA | 2 | 2 | 2 | 2 | 0 | 2 | 2 | 0 |
| Larrain (2001) | 2 | 2 | 2 | 1 | 2 | 2 | 0 | 0 | 0 | 2 | 0 | 0 |
| Kim  (2011) | 2 | 2 | 2 | 2 | 2 | 2 | 2 | 2 | 2 | 2 | 0 | 0 |
| Arciero (2001) | 2 | 2 | 2 | 2 | 2 | 2 | 2 | 2 | 0 | 2 | 0 | 0 |
| McLeod (2021) | NA | NA | NA | NA | 2 | 2 | 2 | 2 | 0 | 2 | 1 | 0 |
| Gigis (2014) | 2 | 2 | 2 | 2 | 2 | 0 | 2 | 2 | 0 | 2 | 0 | 0 |

*Supplemental table 3: Risk of bias for non-randomised studies using the MINORS criteria; 0 = not reported, 1 = reported and inadequate, 2 = reported and adequate. NA used for first 4 criteria if study is non-comparative.*

*Supplemental figure 2: MINORS risk of bias criteria presented by category for all non-randomised studies*
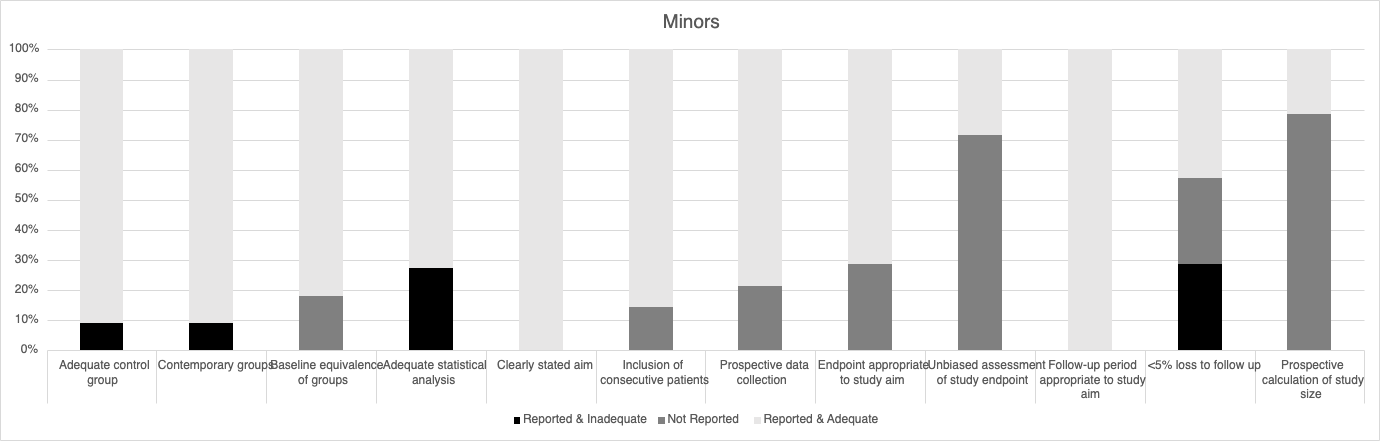

Supplement: sj-docx-1-sel-10.1177_17585732241254693 - Supplemental material for A systematic review and meta-analysis of operative versus non-operative management for first time traumatic anterior shoulder dislocation in young adults [file sj-docx-1-sel-10.1177_17585732241254693.docx]
